# The Accuracy of the Passive Leg Raising Test Using the Perfusion Index to Identify Preload Responsiveness—A Single Center Study in a Resource-Limited Setting

**DOI:** 10.3390/diagnostics15010103

**Published:** 2025-01-04

**Authors:** Marialessia Casazzo, Luigi Pisani, Rabiul Alam Md Erfan Uddin, Abdus Sattar, Rashed Mirzada, Abu Shahed Mohammad Zahed, Shoman Sarkar, Anupam Barua, Sujat Paul, Mohammad Abul Faiz, Abdullah Abu Sayeed, Stije J. Leopold, Sue J. Lee, Mavuto Mukaka, Mohammed Abul Hassan Chowdhury, Ketsanee Srinamon, Marja Schilstra, Asok Kumar Dutta, Salvatore Grasso, Marcus J. Schultz, Aniruddha Ghose, Arjen Dondorp, Katherine Plewes

**Affiliations:** 1 Department of Anesthesia and Intensive Care, University of Bari, 70124 Bari, Italy; marialessia.casazzo@gmail.com (M.C.); salvatore.grasso@uniba.it (S.G.); 2 Mahidol–Oxford Tropical Medicine Research Unit (MORU), Mahidol University, Bangkok 10400, Thailand; sue@tropmedres.ac (S.J.L.); mavuto@tropmedres.ac (M.M.); ketsanee@tropmedres.ac (K.S.); marjaschilstra@yahoo.com (M.S.); marcus.jschultz@gmail.com (M.J.S.); arjen@tropmedres.ac (A.D.); katherine@tropmedres.ac (K.P.); 3 Centre for Tropical Medicine and Global Health, Nuffield Department of Medicine, University of Oxford, Oxford OX3 7LG, UK; 4 Department of Medicine, Chittagong Medical College Hospital, Chattogram 4203, Bangladesh; rabi55dmc@hotmail.com (R.A.M.E.U.); dr.sattar_cox@yahoo.com (A.S.); drrashedmirjada69@gmail.com (R.M.); drasmzahed9a@gmail.com (A.S.M.Z.); drshmn73@gmail.com (S.S.); anupambarua63@yahoo.com (A.B.); sujatpaul123@yahoo.com (S.P.); abdullahdr25@yahoo.com (A.A.S.); asokdutta@yahoo.com (A.K.D.); anrdghs@yahoo.com (A.G.); 5 Malaria Research Group & Dev Care Foundation, Dhaka 1209, Bangladesh; drmafaiz@gmail.com; 6 Department of Internal Medicine, Amsterdam University Medical Centers, Location AMC, 1105 AZ Amsterdam, The Netherlands; stijeleopold@gmail.com; 7 Department of Infectious Diseases, The Alfred Hospital and Central Clinical School, Monash University, Melbourne, VIC 3004, Australia; 8 Bangladesh Institute for Tropical Infectious Diseases, Chattogram 4217, Bangladesh; dr.mahassan09@yahoo.com; 9 Department of Intensive Care, Amsterdam University Medical Centers, Location AMC, 1105 AZ Amsterdam, The Netherlands; 10 Department of Anesthesia, General Intensive Care and Pain Management, Division of Cardiothoracic and Vascular Anesthesia & Critical Care Medicine, Medical University of Vienna, 1090 Vienna, Austria; 11 Department of Global Health, Amsterdam University Medical Centers, Location AMC, 1005 AZ Amsterdam, The Netherlands; 12 Division of Infectious Diseases, Department of Medicine, University of British Columbia, Vancouver, BC V5Z 1L5, Canada

**Keywords:** fluid responsiveness, hypoperfusion, sepsis, malaria, passive leg raising, PLR, perfusion index, PI, low-resource settings, resource limitations

## Abstract

**Background:** We investigated the accuracy of predicting preload responsiveness by means of a passive leg raising test (PLR) using the perfusion index (PI) in critically ill patients showing signs of hypoperfusion in a resource-limited setting. **Methods:** We carried out a prospective observational single center study in patients admitted for sepsis or severe malaria with signs of hypoperfusion in Chattogram, Bangladesh. A PLR was performed at baseline, and at 6, 24, 48, and 72 h. Preload responsiveness assessed through PI was compared to preload responsiveness assessed through cardiac index (CI change ≥5%), as reference test. The primary endpoint was the accuracy of preload responsiveness prediction of PLR using PI at baseline; secondary endpoints were the accuracies at 6, 24, 48, and 72 h. Receiver operating characteristic (ROC) curves were constructed. **Results:** The study included 34 patients admitted for sepsis with signs of hypoperfusion and 10 patients admitted for severe malaria. Of 168 PLR tests performed, 143 had reliable PI measurements (85%). The best identified PI change cutoff to discriminate responders from non–responders was 9.7%. The accuracy of PLR using PI in discriminating a preload responsive patient at baseline was good (area under the ROC 0.87 95% CI 0.75–0.99). The test showed high sensitivity and negative predictive value, with comparably lower specificity and positive predictive value. Compared to baseline, the AUROC of PLR using PI was lower at 6, 24, 48, and 72 h. Restricting the analysis to sepsis patients did not change the findings. **Conclusions:** In patients with sepsis or severe malaria and signs of hypoperfusion, changes in PI after a PLR test detected preload responsiveness. The diagnostic accuracy was better when PI changes were measured at baseline.

## 1. Introduction

Fluid therapy is a powerful resuscitation method but can cause substantial harm [1,2]. The Surviving Sepsis Campaign guidelines advise against liberal fluid administration in patients with sepsis [3,4,5], while the World Health Organization guidelines for the treatment of malaria advise restrictive and cautious fluid therapy [6,7]. Despite the different etiology of impaired tissue perfusion, both patients with severe malaria and sepsis are at an increased risk of organ dysfunction exacerbated by over or under fluid resuscitation, leading to poorer outcomes. Only half of critically ill patients with hypoperfusion actually respond to fluid therapy, as determined by significant changes in cardiac output in response to a fluid bolus [8]. A passive leg raising (PLR) test could help identify patients that may be fluid responsive [9].

A PLR test mandates real-time measurement of cardiac index (CI), e.g., by means of hemodynamic monitoring tools or echocardiography that are infrequently available and often not feasible in resource-limited settings. The perfusion index (PI) [10] may serve as an attractive alternative to cardiac output measurements [10,11]. The PI is derived from relatively inexpensive and widely available pulse oximeters [12]. The PI represents the ratio between two components of the plethysmographic oxygen saturation curve: the pulsatile part reflects the arterial blood volume changes in the finger and non-pulsatile portion is related to other tissue, such as connective tissue, bone, and venous blood. Changes in the PI value, assuming that the non-pulsatile portion is not influenced by other aspects, would then mirror changes in the stroke volume and consequently in the cardiac output, as shown after fluid loading [10,11], PLR [10,11], recruitment maneuvers [13], end-expiratory occlusion tests [11], and tidal volume challenges [14]. All studies were conducted in high-resource settings and mostly in mechanically ventilated patients. It is uncertain if fluid responsiveness prediction by means of PLR determined by PI is as accurate compared to PLR determined by CI.

We assessed the diagnostic accuracy of fluid responsiveness prediction of PLR tests assessed through PI in patients with hypoperfusion due to sepsis or severe malaria in a resource-limited setting. We hypothesized that preload responsiveness assessed by PI performs well in these patients.

## 2. Methods

### 2.1. Design and Ethical Approval

This was a prospective observational single center study in critically ill patients admitted to a medical ward, high-dependency unit, or intensive care unit of the Chittagong Medical College Hospital (CMCH) in Chattogram, Bangladesh, named the ‘Perfusion and lung congestion Evaluation Related to Fluids and vasopressors in Sepsis and malaria study’ (PERFuSE). The study was conducted between 4 June 2019 and 27 August 2019.

The study protocol was approved by the Oxford University Tropical Research Ethics Committee (OxTREC reference number 11–18), the Bangladesh Medical Research Council Ethics Committee (approval number BMRC/NREC72016-2019/798), and the Chittagong Medical College Ethical Review Committee (approval number CMC/PG/2018/51). The study had several research questions and was registered on clinicaltrials.gov (NCT03641534). Written informed consent was obtained from each patient or legally acceptable representative if the patient was unconscious or aged below 16 years.

### 2.2. Patient Characteristics

Patients were eligible for participation in PERFuSE if the following were satisfied: (1) admitted to hospital for sepsis with signs of hypoperfusion (systolic blood pressure (SBP) ≤ 100 mmHg), or receiving vasopressor (epinephrine, norepinephrine, dopamine) plus at least one of respiratory rate (RR) ≥ 22 breaths per minute or receiving oxygen therapy or mechanical ventilation or altered mental status (Glasgow Coma Scale ≤ 14); or (2) confirmed malaria cases.

Patients were excluded if they were younger than 12 years of age, declined informed consent, or had a known malignancy or chronic liver disease, a recent surgery, or trauma resulting in the current hospital admission. In addition, patients with uncomplicated malaria were excluded from this analysis, i.e., patients with asexual parasitemia (*P. falciparum* or *P. vivax*) missing at least one severity criterion among: Glasgow coma scale < 11; hematocrit < 20% with parasite count > 100,000/mm^3^; jaundice with parasite count > 100,000/mm^3^; serum creatinine > 3 mg/dL (or anuria); hypoglycemia with venous glucose < 40 mg/dL systolic blood pressure < 80 mmHg with cool extremities; peripheral asexual stage parasitemia > 10%; peripheral venous lactate > 4 mmol/L or peripheral venous bicarbonate < 15 mmol/L; respiratory distress, radiologically confirmed pulmonary edema, or oxygen saturation < 92% in room air with a respiratory rate of more than 30 breaths per minute, with chest indrawing or crepitations on auscultation; spontaneous bleeding; generalized convulsions (≥2 episodes in 24 h).

### 2.3. Data Collected

We collected a full medical history and performed a physical examination. At enrolment, venous blood samples were analyzed for electrolytes, glucose, pH, and bicarbonate using a bedside analyzer (iSTAT, Abbott, IL, USA). Patients were followed until hospital discharge or death in the hospital. Follow up after hospital discharge occurred on day 14 and day 30 from enrolment.

### 2.4. PLR

A PLR was performed at baseline and at 6, 24, 48, and 72 h, using a manual raise of the legs from a supine position to 45° [8,9,10,15]. In case of known contraindications to the PLR maneuver (i.e., intra-cranial or intra-abdominal hypertension), the test was not performed and the reason was reported. The hemodynamic measures were recorded 60 to 90 s after completing the manual raise of the legs.

The PLR test could also be interrupted in cases of patient discomfort or based on treating team requests. Catecholamine and sedative medication dosages as well as ventilation settings were kept constant during the PLR. A PLR test was defined as positive if a rise in CI of 5% or more was observed [15].

### 2.5. Cardiac Index, Perfusion Index, and Hemodynamic Parameters

The CI was derived through transthoracic echocardiography (LOGIQ V2 apparatus, GE Healthcare, Chalfont St. Giles, UK) as described previously [16]. Left ventricular outflow tract diameter (LVOTd) was measured from parasternal long axis images at baseline. Left ventricular outflow tract velocity time integral (VTI) was measured by pulsatile Doppler LVOT flow pattern in an apical 5-chamber window. Body surface area (BSA) was calculated using the DuBois formula.

The PI was calculated automatically from a MightySat pulse oximeter (Masimo, Irvine, CA, USA) as the ratio between the amplitude of the pulsatile and non-pulsatile blood flow components and expressed as a percentage. The device displays the PI value in real time with no averaging. The PI value was recorded when the displayed value was stable for more than five seconds.

Mean arterial pressure was calculated as diastolic blood pressure + 0.01 * exp (4.14–40.74/Heart rate) * (systolic blood pressure—diastolic blood pressure). The maximum inferior vena cava diameter was measured using M-mode 1.5–2 cm from the right atrium.

### 2.6. Endpoints

The primary endpoint was the accuracy of PLR tests assessed through PI to detect preload responsiveness. The secondary endpoint was their accuracy at subsequent time points.

### 2.7. Power Calculation

There was no formal sample size calculation; all eligible patients with a reliable PI signal were considered for inclusion in this study. A post hoc power calculation was performed: assuming a 60% positivity for preload responsiveness, and an AUC of 92% ± 10%, with 95% confidence interval, 30 patients would be required.

### 2.8. Statistical Analysis

Data are expressed as mean ± standard deviation, median with interquartile range (IQR), or numbers and percentages, as appropriate. PLRs with missing data were excluded from the analysis, i.e., in case of a contraindication to the maneuver, missed PLR tests, patient discharge before the set time point, unreliable PI data, or interrupted PLR tests. We also excluded PLRs with an unreliable reference test. The diagnostic ability of the PI was compared against the change in CI at a cutoff of 5% as the gold standard [17]. Receiver operating characteristic (ROC) curves were constructed and the area under the ROC (AUROC) calculated. The optimal cutoff value for prediction of preload response by PI was derived with the data from all the time points combined, while diagnostic accuracy metrics were reported for each separate time point. In order to select the best cutoff, the optimal cutoff points were found by maximizing the product of the sensitivity and specificity (Liu’s method), maximizing the sum of sensitivity and specificity (Youden method), and by finding the point on the ROC curve nearest to (0,1). The cut point that resulted in the highest negative predictive value was thus selected. The accuracy metrics calculated included sensitivity, specificity, and the positive predictive value (PPV) and negative predictive value (NPV). An AUROC of ≥0.90 was considered excellent, 0.80 to 0.89 was considered good, 0.70 to 0.79 was considered moderate, 0.60 to 0.69 was considered poor, and <0.60 was considered a fail [18].

In one sensitivity analysis, we restricted the analysis to patients with sepsis.

All statistical analyses were performed in Stata Statistical Software, (release 17.0, StataCorp LLC, College Station, TX, USA). A P of 0.05 was considered statistically significant.

## 3. Results

### 3.1. Patients and PLR Tests

**Figure 1:**
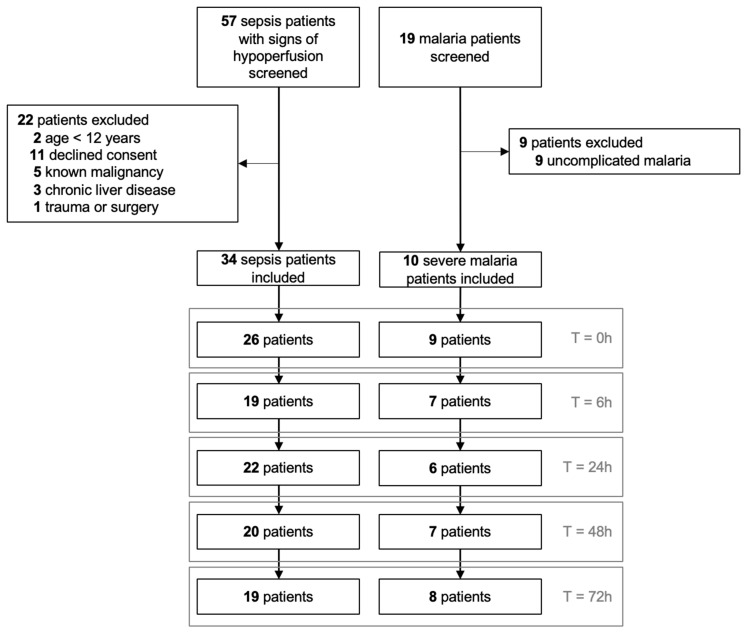
Patient flow, with the numbers of patients at which PLR was performed at successive time points.

Patients were screened from 4 June to 27 August 2019. Of 57 eligible patients, 44 patients were enrolled; 34 patients were admitted for sepsis and 10 patients for severe malaria (Figure 1). Nearly half of the patients received vasopressors, but a minority of patients were intubated for invasive ventilation (Table 1). Overall hospital mortality was 27% for the entire cohort. Not all planned PLRs could be performed due to various practical reasons. Out of 179 PLR tests initiated, 106/136 (78%) in sepsis patients and 37/43 (86%) in severe malaria patients had complete data and were included in this analysis. Details of PLRs at hour 0 are presented in Table 2 and Table S2. Changes in CI and PI were more pronounced in responders as compared to non-responders. Patients’ heart rate remained stable before and after PLR tests. The median maximum IVC diameter was <20 mm in both responders and non-responders. Patients with severe malaria were more often preload responsive as compared to sepsis patients (Table S2).

### 3.2. Accuracy of PLR Assessed by PI at Baseline

**Figure 2:**
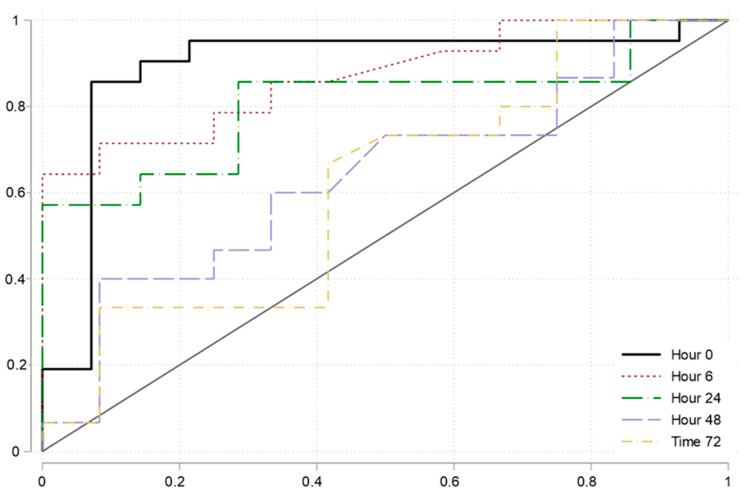
ROC curves showing the accuracy of the passive leg raising test using the perfusion index at various time points.

At baseline, 21/35 (60%) patients had a positive PLR assessed by the reference test. The best identified PI change cutoff to discriminate responders from non-responders was 9.7%. The accuracy of PLR assessed by PI in discriminating a preload responsive patient at baseline was good (AUROC, 0.87 [95%–CI 0.75 to 0.99]) (Figure 2 and Table 3). The sensitivity of PLR assessed by PI was 95.2 (76.2–99.9), the specificity was 78.6 (49.2–95.3), the PPV was 87.0 (66.4–97.2), and the NPV 91.7 (61.5–99.8).

### 3.3. Accuracy of PLR Assessed by PI at Successive Time Points

Preload responsiveness according to the reference test remained stable (50–56%). Compared to baseline, the accuracy of PLR assessed by PI in discriminating a preload responsive patient at baseline declined (Figure 2 and Table 3). In accordance, sensitivity, specificity, PPV, and NPV declined as well.

### 3.4. Sensitivity Analysis

For sepsis patients, 50% of preload responsiveness was found at hour 0. The AUROC for the PLR assessed by PI in discriminating preload responsive patients was 0.89 [0.77 to 1.0], with similar accuracy metrics to the whole group (Table 3).

## 4. Discussion

This prospective observational study investigated the accuracy of the PLR test assessed by PI in a resource-limited setting. The findings can be summarized as follows: (a) the accuracy of PLR assessed by PI at baseline was good; (b) a cutoff of 10% maximized negative predictive value. However, (c) the accuracy decreased at successive time points.

Our findings are in line with previous studies, demonstrating the accuracy of PI in detecting preload responsiveness. The finding that the effectiveness of PI in detecting preload responsiveness was good aligns with conclusions from an earlier study conducted in high-resource settings using PI measurements alongside standard PLR maneuvers [10]. Notably, the best cutoff in our study was nearly identical to the one identified previously [10]. Additionally, a recent study demonstrated how PI can be used as a reliable surrogate of CI for testing preload responsiveness using the end-expiratory occlusion test, albeit with a much smaller cutoff of 2.5% [11]. Our results suggest that PI has potential for routine clinical application, especially in identifying patients who are unlikely to benefit from early fluid administration and may even be harmed by it [19]. Pulse oximeters such as the one used in this study to determine PI variations after PLR tests are highly portable and have a low cost. This would allow for routine use in emergency departments before ward admission or in prehospital settings, preventing potentially harmful fluid administration.

Several factors may contribute to the decline in performance observed at subsequent time points. Various elements influence the plethysmographic signal beyond the CI. For instance, a decrease in venous blood flow due to worsening septic vasoplegia may elevate the non-pulsatile component of PI, thereby dampening PI regardless of stroke volume [10]. Moreover, the accumulation of peripheral tissue edema, commonly seen in patients with sepsis and severe malaria patients with kidney failure, can degrade the PI signal, resulting in decreased accuracy. Previous evidence on the potential for the PI to reliably detect changes in arterial flow has been non-definitive. Changes in PI or in pulse oximeter waveform were found to reflect changes in cardiac index [10,20] or in the amplitude of arterial pressure [20] in several settings. Fluid-induced changes in cardiac index could be detected by a change in pulse oximeter waveform even after anesthesia induction [21]. Yet, another study did not find a significant change in PI during a PLR maneuver [20], while another reported modest agreement in the operating theater [21]. While these findings advise caution in using PI days after baseline, they do not hinder the potential benefits of its early application.

The PLR test is a validated technique for predicting fluid responsiveness in patients with confirmed or suspected low cardiac output [22,23,24]. However, a key limitation of PLR tests is the necessity of a direct measure of stroke volume to detect short-term changes in blood flow [24]. While other simple metrics, such as pulse pressure variation, have been explored for studying PLR effects, their sensitivity has been found inadequate [9,25]. In our study, divergent results were observed with PLR assessed by PI, which showed high sensitivity but lower specificity. Therefore, in settings lacking direct measurements of CI, the PI might effectively be combined pulse pressure variations assessed with a simple blood pressure monitor, accurately excluding patients unlikely to benefit from fluid administration.

In resource-limited settings, the ability to perform a conventional semirecumbent PLR maneuver is limited due to the scarcity of tiltable beds. This limitation can be mitigated by manual supine PLRs performed by healthcare providers, even if limited data exist on their comparative efficacy [15]. The supine PLR consists of solely elevating the legs, instead of tilting the bed. However, this method may mobilize an insufficient quantity of blood and inadequately challenge the heart [15]. Nevertheless, the exact lower CI cutoff to use for manual PLRs maneuvers as compared to PLRs using a tiltable bed was not established, hence the arbitrary choice for 5% in our study. On the other hand, the observed stability in heart rate during PLR tests in our study indicates the high-quality of the tests. There is a pressing need to validate simple tools for personalized hemodynamic assessments in settings lacking tiltable beds, or even with patients lying on hospital floors, as was often the case in this study setting [26,27].

Strengths of this study include a rigorous screening methodology and a prospective observational design implementing predefined standard operating procedures. Various predefined time points were explored, thus systematically exploring the first days from sepsis or malaria diagnosis. We also assessed two distinct patient groups of critically ill patients at risk of hypoperfusion, both very common in low- and middle-income countries.

Our study also has limitations. Due to the observational nature of the study, patients did not systematically receive fluids based on the PLR results, precluding verification of actual fluid responsiveness. Comparing supine PLR and semirecumbent PLR maneuvers was not feasible due to the shortage of tiltable beds. It was also not possible to capture dosages of vasoconstrictor drugs known to affect the plethysmographic signal. The inclusion of only sepsis and severe malaria patients restricts the generalizability of our findings to broader patient cohorts. Last but not least, the number of severe malaria patients in our study was low, limiting any inference regarding this specific group of patients.

## 5. Conclusions

We found a good diagnostic accuracy of preload responsiveness prediction at baseline of PLRs using PI. Specifically, our results suggest that PI has potential for routine clinical application in resource-limited settings, especially in identifying patients who are unlikely to benefit from early fluid administration and may even be harmed by it. However, the accuracy of PLR tests assessed by PI at successive time points decreases, which supports exercising caution in using PI after baseline.

## Figures and Tables

**Table 1:** Patient demographics, baseline characteristics, and outcome.

	All (*n* = 44)	Sepsis (*n* = 34)	Severe Malaria (*n* = 10)
Age (years), median (IQR)	37.5 (23, 52)	46 (26, 55)	27 (21, 35)
Female sex, *n* (%)	22 (50%)	20 (59%)	2 (20%)
BMI (kg/m^2^), mean (SD)	22.4 (3.4)	22.4 (3.4)	22.1 (3.6)
SOFA score, median (IQR)	6 (3, 10)	5 (3, 8)	9 (7, 10)
qSOFA, median (IQR)	2 (2, 2)	2 (2, 2)	2 (2, 2)
Sepsis source *			
Meningitis	-	3 (9%)	-
Pulmonary	-	7 (20%)	-
Hepatobiliary	-	4 (12%)	-
Gastrointestinal	-	13 (38%)	-
Urinary	-	6 (18%)	-
Other	-	3 (9%)	-
RR ≥ 22 bpm, *n* (%)	44 (100%)	34 (100%)	10 (100%)
GCS < 15, *n* (%)	18 (41%)	11 (32%)	7 (70%)
SBP < 100 mmHg, *n* (%)	32 (73%)	29 (85%)	3 (30%)
Use of vasopressors, *n* (%)	18 (40%)	16 (47%)	1 (10%)
Number of malaria severity criteria, median (IQR)	-	-	3 (2, 4)
Parasites/μL, geometric mean (95% CI)	-	-	44,814 (3755, 534,881)
Bilateral pitting edema, *n* (%)	2 (5%)	1 (3%)	1 (10%)
Mechanical ventilation, *n* (%)	2 (5%)	1 (3%)	1 (10%)
Venous base excess (mmol/L), mean (SD)	−5.9 (7.2)	−4.8 (6.8)	−9.7 (7.7)
Venous lactate level (mmol/L), median (IQR)	2.5 (1.2, 4.3)	2.3 (1.2, 4.1)	3.6 (2.2, 8.9)
WBC count, (×10^3^/μL), geometric mean (95% CI)	11.3 (9.0, 14.2)	11.2 (8.6, 14.6)	11.7 (6.6, 20.6)
Hospital mortality, *n* (%)	12 (27%)	10 (29%)	2 (20%)

Abbreviations: BMI, body mass index; qSOFA, quick sequential organ failure assessment; RR, respiratory rate; GCS, Glasgow coma scale; SBP, systolic blood pressure; IQR, interquartile range; SD, standard deviation; WBC, white blood cell. * Patients could have more than one suspected source of sepsis.

**Table 2:** Hemodynamic parameters, before and after a supine passive leg raise test in preload responders and preload non-responders at hour 0.

	Responders (*n* = 21)		Non-Responders (*n* = 14)	
	Baseline	After PLR	Baseline	After PLR
**Reference test**				
Cardiac index (L/min/m^2^)	3.2 (2.7–3.8)	3.8 (3.0–4.4)	3.4 (2.7–4.1)	3.3 (2.6–3.9)
**Index test**				
Perfusion index (%)	3.7 (1.8–10)	6.8 (3.3–11.0)	4.8 (1.9–9.5)	5.7 (1.7–10.4)
**Other parameters**				
Heart rate (beats/minute)	101 (84–114)	103 (85–113)	100 (87–111)	98 (86–111)
Systolic BP (mmHg)	101 (93–113)	103 (96–114)	98 (90–111)	99 (92–114)
Diastolic BP (mmHg)	63 (55–71)	65 (57–71)	65 (56–70)	66 (57–72)
MAP (mmHg)	78 (71–88)	81 (74–88)	78 (71–86)	80 (72–86)
Capillary refill time (sec)	1.8 (1–2)	-	2.0 (1–2)	-
Maximum IVC diameter (mm)	13 (10–16)	-	14 (11–16)	-

All values reported as median (IQR). Abbreviations: PLR, passive leg raise test; IQR, interquartile range; BP, blood pressure; MAP, mean arterial pressure; BP, blood pressure; IVC, inferior vena cava.

**Table 3:** Diagnostic accuracy measures assessing whether changes in perfusion index discriminate fluid-responsive patients according to reference cardiac index method.

Time Point	*n*	Responders %	TP	TN	FP	FN	AUROC (95% CI)	Sensitivity % (95% CI)	Specificity % (95% CI)	PPV % (95% CI)	NPV % (95% CI)
**All Patients**											
0 h	35	60%	20	11	3	1	0.87 (0.75–0.99)	95.2 (76.2–99.9)	78.6 (49.2–95.3)	87.0 (66.4–97.2)	91.7 (61.5–99.8)
6 h	26	54%	11	8	4	3	0.73 (0.55–0.91)	78.6 (49.2–95.3)	66.7 (34.9–90.1)	73.3 (44.9–92.2)	72.7 (39.0–94.0)
24 h	28	50%	12	10	4	2	0.79 (0.63–0.94)	85.7 (57.2–98.2)	71.4 (41.9–91.6)	75.0 (47.6–92.7)	83.3 (51.6–97.9)
48 h	27	56%	11	6	6	4	0.62 (0.43–0.80)	73.3 (44.9–92.2)	50 (21.1–78.9)	64.7 (38.3–85.8)	60 (26.2–87.8)
72 h	27	56%	9	7	5	6	0.59 (0.40–0.79)	60 (32.3–83.7)	58.3 (27.7–84.8)	64.3 (35.1–87.2)	53.8 (25.1–80.8)
**Sepsis Patients**											
0 h	26	50%	13	10	3	0	0.89 (0.77–1.0)	100 (75.3–100)	76.9 (46.2–95)	81.3 (54.4–96.0)	100 (69.2–100)
6 h	19	47%	6	6	4	3	0.63 (0.40–0.86)	66.7 (29.9–92.5)	60.0 (26.2–87.8)	60.0 (26.2–87.8)	66.7 (29.9–92.5)
24 h	22	41%	8	9	4	1	0.79 (0.62–0.96)	88.9 (51.8–99.7)	69.2 (38.6–90.9)	66.7 (34.9–90.1)	90.0 (55.5–99.7)
48 h	20	50%	8	5	5	2	0.65 (0.44–0.86)	80.0 (44.4–97.5)	50.0 (18.7–81.3)	61.5 (31.6–86.1)	71.4 (29.0–96.3)
72 h	19	53%	6	4	5	4	0.52 (0.29–0.76)	60.0 (26.2–87.8)	44.4 (13.7–78.8)	54.5 (23.4–83.3)	50.0 (15.7–84.3)

Abbreviations: TP, true positive case; TN, true negative case; FP, false positive case; FN, false negative case; PPV, positive predictive value; NPV, negative predictive value; AUROC, area under the receiver operating characteristics curve.

## Data Availability

Original data are available upon reasonable request to the corresponding author(s).

## Supplementary Materials

The following supporting information can be downloaded at: https://www.mdpi.com/article/10.3390/diagnostics15010103/s1, Table S1: STROBE checklist; Table S2: Hemodynamic parameters at hour 0, before and after a supine PLR stratified by response to PLR and by patient group (sepsis and severe malaria); Figure S1: Patient flow, with the numbers of patients at which PLR was performed at successive time points and detailed reasons of exclusion or missing data.
